# Effectiveness of a Self-Monitoring App in Supporting Physical Activity Maintenance Among Rural Canadians With Cancer After an Exercise Oncology Program: Cluster Randomized Controlled Trial

**DOI:** 10.2196/47187

**Published:** 2023-09-07

**Authors:** Manuel Ester, Chad W Wagoner, Julianna Dreger, Guanmin Chen, Meghan H McDonough, Margaret L McNeely, S Nicole Culos-Reed

**Affiliations:** 1 Faculty of Kinesiology University of Calgary Calgary, AB Canada; 2 Department of Community Health Sciences University of Calgary Calgary, AB Canada; 3 Data and Analytics Alberta Health Services Calgary, AB Canada; 4 Department of Physical Therapy University of Alberta Edmonton, AB Canada; 5 Department of Oncology University of Alberta Edmonton, AB Canada; 6 Rehabilitation Medicine Cross Cancer Institute Edmonton, AB Canada; 7 Department of Oncology Cummings School of Medicine University of Calgary Calgary, AB Canada; 8 Department of Psychosocial Resources Tom Baker Cancer Centre Alberta Health Services Cancer Care Calgary, AB Canada

**Keywords:** eHealth, mHealth, mobile health, mobile apps, self-monitoring, cancer, oncology, physical activity, exercise, randomized controlled trial, intervention, mobile phone

## Abstract

**Background:**

Despite the benefits of physical activity (PA) for individuals with cancer, most remain insufficiently active. Exercise oncology interventions can improve PA levels. Individuals struggle to maintain PA levels after interventions because of persistent psychological and environmental PA barriers. Health technology (eHealth) may address some PA barriers and deliver effective, scalable PA interventions in oncology, yet its effectiveness for changing PA levels remains mixed. Using eHealth to support PA maintenance among rural populations with cancer, who may need greater PA support given lower PA levels and worse health outcomes, remains under-studied.

**Objective:**

This study examined the effectiveness of an app-based self-monitoring intervention in supporting PA maintenance among rural populations with cancer after a supervised web-based exercise oncology program.

**Methods:**

This 2-arm, cluster randomized controlled trial was embedded within the Exercise for Cancer to Enhance Living Well (EXCEL) effectiveness-implementation study. Upon consent, participants were randomized 1:1 by EXCEL class clusters to the intervention (24 weeks of app-based PA self-monitoring) or waitlist control (app access after 24 weeks). Both groups completed a 12-week supervised web-based exercise oncology program followed by a 12-week self-directed PA maintenance period. Baseline demographics, eHealth literacy, and patient-reported outcomes were compared using chi-square and 2-tailed *t* tests. App use was measured throughout the intervention. The primary outcome—self-reported moderate-to-vigorous PA (MVPA) minutes—and secondary outcomes—objective MVPA minutes and steps and app usability ratings—were collected at baseline, 12 weeks, and 24 weeks. Intervention effects on self-report MVPA maintenance were assessed via linear mixed modeling, with secondary outcomes explored descriptively.

**Results:**

Of the 359 eligible EXCEL participants, 205 (57.1%) consented, 199 (55.4%; intervention: 106/199, 53.3%; control: 93/199, 46.7%) started the study, and 183 (51%; intervention: 100/183, 54.6%; control: 83/183, 45.4%) and 141 (39.3%; intervention: 69/141, 48.9%; control: 72/141, 51.1%) completed 12- and 24-week measures, respectively. Mean age was 57.3 (SD 11.5) years. Most participants were female (174/199, 87.4%), White (163/199, 81.9%), and diagnosed with breast cancer (108/199, 54.3%). Median baseline self-report weekly MVPA minutes were 60.0 (IQR 0-180) and 40.0 (IQR 0-135) for the intervention and waitlist control groups, respectively (*P*=.74). Median app use duration was 10.3 (IQR 1.3-23.9) weeks, with 9.6 (IQR 4.4-17.8) self-monitoring entries/week. Both groups increased their weekly MVPA minutes significantly at 12 weeks (*P*<.001) and maintained the increases at 24 weeks (*P*<.001), relative to baseline, with no between-group differences (*P*=.87). The intervention group had significantly higher step counts for 7 of the 12 weeks during the PA maintenance period (*P*=.048 to <.001).

**Conclusions:**

The app-based self-monitoring intervention did not improve MVPA maintenance but may have contributed to increased step counts during the PA maintenance period. More work is needed to realize the full potential of eHealth in exercise oncology.

**Trial Registration:**

ClinicalTrials.gov NCT04790578; https://clinicaltrials.gov/study/NCT04790578

**International Registered Report Identifier (IRRID):**

RR2-10.1016/j.cct.2021.106474

## Introduction

### Background

Physical activity (PA) can improve physical function, fatigue, mental health (anxiety and depression), and the overall quality of life (QoL) among populations with cancer [1,2]. However, despite these benefits and emerging efforts to increase PA levels in oncology, recent cross-sectional data show that only 12% of individuals living with and beyond cancer meet guideline recommendations for weekly PA, with below-average PA levels for rural individuals compared with their urban counterparts [1,3-6]. Although supervised in-person interventions show promise for increasing PA levels and improving QoL in oncology, systems-level (eg, cost, the lack of resources, and environmental impacts such as COVID-19) and individual-level (eg, lack of time and access to facilities) barriers, which are often exacerbated in rural and remote areas, have limited their implementation and impact to date [7]. Furthermore, most prior studies on exercise oncology interventions examined short-term interventions lasting up to 3 months and focused primarily on the initial adoption phase of PA behavior change [8].

Although it is crucial to sustain the positive impacts of exercise oncology interventions, PA maintenance—supporting individuals to stay active in the long term—remains a key challenge. According to the transtheoretical model of behavior change, maintenance is defined as sustained behavior change for 6 months after adoption, with a recent review in exercise oncology suggesting that PA levels 3 months after the intervention provide a good indicator of PA maintenance [9,10]. Even after starting exercise behavior change in a supervised exercise oncology program, participants may still face significant PA maintenance barriers (eg, the lack of motivation, confidence, access to exercise facilities, and time), and PA levels thus tend to decline rapidly after a formal program ends [10-12]. Given these challenges and the importance of PA maintenance, further research on how to support PA maintenance in exercise oncology is warranted.

To address the existing challenges to understanding PA maintenance in exercise oncology, research has begun to examine health technology–based (eHealth) exercise oncology interventions [13,14]. Interventions delivered via eHealth, including mobile technologies (mobile health, eg, apps and wearables) and others (eg, videoconferencing and websites), may be able to address some of the systems-level and individual-level barriers to PA maintenance [15]. For example, self-directed eHealth PA interventions can be less resource intensive than supervised in-person PA interventions [16]. Furthermore, they have been shown to increase motivation and confidence while reducing time and access barriers to individual PA participation in both healthy adults and populations with cancer [17,18]. Surveys of populations with cancer indicate high levels of interest in eHealth PA interventions; high use of technology such as smartphones and computers; and positive perceptions of the usefulness of mobile health, specifically to support PA habits [19-22]. However, research to date has shown only mixed effectiveness of eHealth exercise oncology interventions in increasing PA [23], and less than 20% of interventions to date have measured PA maintenance. Of those that did, only 41% reported positive outcomes on PA maintenance, and none targeted rural and remote populations with cancer [23].

### Study Objective

This study sought to address this knowledge gap by examining the effectiveness of an eHealth intervention in promoting PA maintenance in individuals living with and beyond cancer after their participation in an exercise oncology program. Specifically, this study was embedded within the Exercise for Cancer to Enhance Living Well (EXCEL) 5-year effectiveness-implementation research project. EXCEL provides rural and remote Canadians living with and beyond cancer with a 12-week exercise oncology program featuring twice-weekly group-based exercise classes and integrated PA behavior change education through an *exercise and educate* approach [24,25].

This study’s eHealth intervention was based on a digital journaling mobile app designed to empower users via self-monitoring, a behavior change technique that has been linked to increased effectiveness of PA behavior change interventions in populations with cancer and healthy adults [26,27]. In response to the mixed effectiveness of prior eHealth exercise oncology interventions, multiple rounds of codevelopment with industry partners and individuals living with and beyond cancer were carried out before the study to create a study-specific version of the app to specifically support PA maintenance [19,23,28]. The aim of these primary analyses was to evaluate the effectiveness of the app-based self-monitoring intervention in supporting PA maintenance among rural Canadians living with and beyond cancer after the completion of a supervised web-based exercise oncology program.

## Methods

### Study Design

This paper presents the primary quantitative results of a 2-arm, cluster randomized controlled trial (RCT), which was embedded within the EXCEL effectiveness-implementation study [24,25,29]. This RCT was prospectively registered (ClinicalTrials.gov; NCT04790578). A brief overview of the study is presented below. Additional protocol details for the present RCT and the larger EXCEL project are available elsewhere [24,29].

### Ethical Considerations

Ethics approval was obtained from the Health Research Ethics Board of Alberta’s Cancer Committee (HREBA.CC-20-0283).

All study participants provided informed consent via an electronic form. Study data were deidentified using study ID numbers to ensure participant privacy and confidentiality. No compensation was provided to participants.

### Setting

All components of this study were delivered remotely to participants in rural and remote regions across Canada. Contact with participants occurred via email, Zoom (Zoom Video Communications, Inc) videoconferencing, or a chat function directly in the mobile app.

### Participants and Recruitment

Study participants were required to meet the following eligibility criteria: they should (1) be currently participating in EXCEL exercise oncology classes; (2) have any cancer diagnosis; (3) be aged >18 years; (4) be physically able to participate in mild PA (assessed by a clinical exercise physiologist during prestudy screening); (5) be in pretreatment or on treatment or have completed treatment within the past 3 years; (6) provide written consent in English; (7) have access to internet speeds that support Zoom use; and (8) be located in remote, rural, or underserved (ie, with no exercise oncology resources) areas in Canada.

The study coordinator visited all web-based EXCEL classes during the second week of the 12-week program to provide a study overview, answer questions, and invite participants to join the study. An email invitation with a link to the electronic informed consent form was then sent to participants, with 2 reminders sent at 3-day intervals to those who had not replied. To reach the target sample size, 4 rounds of recruitment were conducted from April 2021 to April 2022, in line with the start times of the 12-week EXCEL exercise oncology program (April 2021, September 2021, January 2022, and April 2022).

### Randomization and Allocation

Upon informed consent, participants were randomized using Sealed Envelope (Sealed Envelope Ltd), a web-based randomization program, to either the app-based self-monitoring intervention or waitlist control group using 1:1 stratified block randomization [30,31]. Randomization by exercise class clusters was performed to improve the integration of the app-based self-monitoring intervention within the group-based EXCEL exercise oncology program by having a class assigned to either include intervention or not, thereby avoiding potential control group contamination within a class. Stratification was done according to class location, with block sizes set according to the number of classes scheduled at each location for each 12-week EXCEL exercise oncology program. The study coordinator (ME) performed the randomization, enrollment, and allocation of participants to groups. The study coordinator was not aware of participants’ baseline measures and had no contact with participants before the recruitment and randomization processes.

### App-Based Self-Monitoring Intervention

#### Overview

The total study duration was 24 weeks, with an initial 12-week EXCEL exercise program period (twice-weekly EXCEL supervised web-based exercise oncology classes), followed by a 12-week PA maintenance period (self-directed PA, ie, participants were encouraged to maintain PA levels) [24]. All participants were enrolled in the 12-week EXCEL exercise program. In addition to the supervised web-based exercise oncology classes, intervention group participants received access to a self-monitoring app for 23 weeks, from week 2 until the end of the 24-week study period. Those in the waitlist control group were able to access the app only after study completion at 24 weeks.

More details about this RCT, including a timeline of the intervention period, screenshots of the app interface, and a complete list of the behavior change techniques (eg, self-monitoring of behavior, prompts and cues, feedback on behavior, and credible source) applied within the study-specific version of the Zamplo app and accompanying support resources, have been previously published [29].

#### App-Based Self-Monitoring

During the second week, intervention group participants received access to a codeveloped study-specific version of Zamplo, a self-monitoring app that could be used via a smartphone or on any device via a web browser [28,29]. Participants were asked to use Zamplo regularly for the remaining 23 weeks of the 24-week study period, including 11 weeks during the EXCEL exercise program period and throughout the subsequent 12-week PA maintenance period, to self-monitor their PA levels and track personally relevant (mental and physical) health outcomes.

Study-specific tracking templates, created by the study team in collaboration with the app developer and individuals living with and beyond cancer, were available on participant home screens upon logging in, each of which could be completed in under 5 minutes [28,29]. The templates included (1) a daily check-in to track total PA, energy, and fatigue; (2) pre– or post–EXCEL exercise class check-ins to track energy, fatigue, and class completion; (3) a weekly check-in for setting a weekly PA goal, recording completion of their previous weekly PA goal, and noting any barriers to and facilitators of achieving the goal; and (4) a monthly check-in featuring the 10-item Edmonton Symptom Assessment System (ESAS) questionnaire [32]. Participants received daily (template 1), biweekly (template 2), weekly (template 3), or monthly (template 4) emails and push notifications to complete these tracking templates.

In addition to using these tracking templates, participants were encouraged to personalize their self-monitoring in Zamplo by adding relevant activities, symptoms (eg, pain and soreness), medications, or other health data (eg, weight and sleep quality) to existing templates or by creating their own templates. For all tracked data, graphs were automatically generated and displayed on the home screen to help participants visualize and reflect on changes in their PA levels and health over time. Graphs could be customized by adding or removing items and changing the colors or format (bar, line, or dotted line) for each item.

#### Additional App Support

Reminders to use Zamplo, instructions on how to customize self-monitoring, and technical support were provided through different tools, as outlined in Table 1.

**Table 1:** Overview of the additional support provided to intervention group participants.

Tool	Details
Notifications	Smartphone, email, and in-app notifications were set up for all study-specific Zamplo journal templates. For user-created templates, participants could choose whether to receive notifications. Participants were shown how to customize notification frequency, timing, and format (smartphone, email, or both) to suit their preferences.
Weekly emails	Weekly emails were sent by the study coordinator (ME) at the start of each week for the first 12 weeks. The emails contained prompts to stick to daily and weekly self-monitoring habits, encouragement to try customizing Zamplo as desired, and a reminder to contact study staff for technical support as needed.
Introductory workshops	Two 1-hour Zoom sessions were hosted during the first 2 weeks of the study to enhance self-efficacy and motivation for using Zamplo. The first workshop focused on the value of self-monitoring for supporting PA habits and interactive demonstrations of basic Zamplo features to help participants with initial learning. The second workshop focused on graphing and customizing Zamplo to individual needs and preferences. Prerecorded versions of both workshops were sent to participants unable to attend, and all participants could revisit content as desired.
Infographic PDF user guides and tutorial videos	Written, verbal, and visual instructions were provided on how to set up, use, and customize Zamplo for self-monitoring during the study.
Ongoing technical support	The study coordinator could be contacted via email or direct messaging in Zamplo in case of any issues, who could help resolve them directly or organize a Zoom support session, if needed. A tracking sheet was used to record details on the type of issue and how it was resolved.

### Protocol Deviations

No changes were made to the intervention after publishing the study protocol [29]. However, a minor change was made to the data collection methods. Given the limited availability of Garmin (Garmin International Inc) devices in EXCEL, not all participants wore the Garmin Vivosmart 4 for collecting objective PA as initially planned. More information on the allocation of Garmin devices is provided in the *Outcomes and Data Collection* section.

### Outcomes and Data Collection

Data were collected at baseline, after the EXCEL exercise program period at week 12, and after the PA maintenance period at week 24. All questionnaires were via the web-based REDCap (Research Electronic Data Capture; Vanderbilt University) system, with data stored securely on the University of Calgary REDCap server [33].

#### Baseline Measures and Exercise Class Attendance

Baseline study measures included self-report demographics, patient-reported psychosocial variables (cognitive function: Functional Assessment of Cancer Therapy-Cognitive Function; FACT-Cog [34]; health-related QoL: Functional Assessment of Cancer Therapy-General; FACT-G [35]; fatigue: Functional Assessment of Chronic Illness Therapy-Fatigue; FACIT-F [36]; and symptom burden: ESAS [32]), prior technology use, perceived usefulness for PA (in-house questionnaire [19]), and eHealth literacy (eHealth Literacy Questionnaire; eHLQ [37]). Responses for the FACT-Cog, FACT-G, and FACIT-F range from 0 (not at all) to 4 (very much) [34-36]. The ESAS items are scored from 0 (best) to 10 (worst) [32]. Finally, the eHLQ scores for individual items range from 1 (strongly disagree) to 4 (strongly agree) [37]. EXCEL exercise class attendance was tracked for the 12-week exercise oncology program period.

#### Measures of Adherence to the Intervention Components

Patterns of app use were collected continuously via the self-monitoring app during the entire 24-week study period. Attendances at the first and second introductory workshops were recorded. In addition, technical issues reported by participants and details of how each issue was resolved were logged by the study coordinator (ME). No a priori cutoffs were defined for the intervention adherence measures.

#### Primary Outcome

The primary outcome of the study was the maintenance of self-report moderate-to-vigorous PA (MVPA) minutes at 24 weeks and 12 weeks after completing the EXCEL exercise oncology program. Weekly MVPA minutes were self-reported via the modified Godin Leisure Time Exercise Questionnaire (m-GLTEQ) at all time points [38]. The m-GLTEQ asked participants to report the frequency and average duration of mild, moderate, strenuous or vigorous, and resistance PAs performed in the past week. Weekly MVPA minutes was selected as the primary PA outcome, as (1) MVPA is a key component of the exercise oncology guidelines [1], and (2) within research using the m-GLTEQ in exercise oncology, MVPA minutes is the most commonly used PA measure and has established validity for use in populations with cancer [38].

#### Secondary and Exploratory Outcomes

Secondary outcomes, which were collected at all time points, included mild aerobic PA and resistance PA minutes measured via the m-GLTEQ [38] and Zamplo app usability and satisfaction measured via the Mobile App Usability Questionnaire (MAUQ) [39]. Responses on the MAUQ range from 1 (strongly disagree) to 7 (strongly agree).

All participants were asked to participate in the objective PA tracking component of EXCEL by wearing a Garmin Vivosmart 4 PA tracker for objective PA measurement. This device was used only for data collection and not as an active component of the intervention. Owing to resource constraints, Garmin devices were not available for all participants; thus, only a subset who consented to wear a Garmin device as part of EXCEL were provided with one. Specifically, the EXCEL study coordinator (JD) provided Garmin devices to participants on a “first-come, first-served” basis according to the number of devices available for the given study period. Objective MVPA minutes and steps measured via Garmin Vivosmart 4 devices were thus included only as exploratory outcomes in this study.

### Data Processing

After extraction from REDCap, all measures were processed and scored according to standard practices for the respective questionnaires. Specifically, the FACT-Cog scores were calculated by summing the responses in each of the 4 subscales (Perceived Cognitive Impairments: 0-72; Impact on QoL: 0-16; Comments from Others: 0-16; and Perceived Cognitive Abilities: 0-28) [34]. The FACT-G was scored using the 4 standard subscales (Physical: 0-28; Social: 0-28; Emotional: 0-24; and Functional: 0-28) and a total score (0-108) [35]. For the FACT-Cog and FACT-G, higher scores indicate higher QoL. The FACIT-F responses were summed to a total score of 0 to 52, with lower scores indicating higher fatigue [36]. Individual symptom scores (0-10) as well as total ESAS symptom burden (0-100) were calculated using the ESAS, with lower scores reflecting lower symptom burden [32]. For each eHLQ domain, the score was calculated by averaging responses across all items belonging to the domain, with the score ranging from 1 (lowest eHealth literacy) to 4 (highest eHealth literacy) [37]. App use summaries were determined via participant Zamplo use logs, which included details on the weeks, days, and minutes used, as well as what was tracked during the study period. The m-GLTEQ self-report PA data were converted to weekly aerobic MVPA (2 × vigorous PA frequency × vigorous PA duration + moderate PA frequency × moderate PA duration), weekly resistance PA minutes (resistance PA frequency × resistance PA duration), and weekly mild aerobic PA minutes (mild PA frequency × mild PA duration) [38]. Mobile app usability was assessed via an overall MAUQ score and scores for each of the 3 MAUQ subscales (MAUQ Ease of Use and Satisfaction; MAUQ System Information Arrangement; and MAUQ Usefulness), calculated by averaging the ratings across all corresponding items, ranging from 0 (low) to 7 (high) [39]. Finally, daily Garmin data summaries were processed to determine the number of valid wear days per week (at least ten hours per day), valid weeks (at least four valid wear days), steps per day and week, and MVPA minutes per day and week [40].

### Sample Size

On the basis of the primary outcome of self-report weekly MVPA minutes during the PA maintenance period and an anticipated mean between-group difference of 60 minutes of MVPA per week, a sample size requirement of 140 participants was determined (70 participants per group, 80% power, 120 min/wk SD, 5% two-tailed α, and 10% attrition) using a web-based tool developed by the Department of Statistics at the University of British Columbia [41,42]. A 60-minute difference was selected based on typical between-group differences seen in previous literature on PA maintenance after exercise oncology interventions and associations between PA levels and clinically meaningful outcomes such as treatment-related side effects, QoL, mortality, and recurrence [10,43].

### Data Analyses

All data were collected and stored in REDCap and exported to RStudio (version 1.3; RStudio, Inc), where the analyses were performed [33]. The data were first explored to examine the nature of missing data and visualize distributions. Descriptive statistics (frequencies and percentages for categorical variables, means and SDs for normally distributed continuous variables, and medians and IQRs for nonnormally distributed continuous variables) were then calculated for baseline characteristics, including demographics, cancer type and treatment, eHealth literacy, technology use, patient-reported outcomes, and self-reported preintervention weekly MVPA minutes. Unpaired 2-tailed *t* tests (continuous and normally distributed), Mann-Whitney *U* tests (continuous and nonnormally distributed), and chi-square tests (categorical) were used to check for between-group differences in the demographics and baseline levels of outcome variables. Descriptive statistics were also calculated for the primary (m-GLTEQ weekly MVPA minutes) and secondary (Garmin MVPA minutes and steps) PA outcomes. Data were then inspected using scatterplots and Pearson correlation coefficients for continuous variables or box plots for categorical variables to examine the relationships between m-GLTEQ weekly MVPA minutes and baseline characteristics. Histograms and residual plots were used to visualize data distributions in preparation for linear mixed modeling. Owing to the skewed nature of the primary outcome (m-GLTEQ weekly MVPA minutes), log-transformed data were used for the analyses to align with the normality assumption in linear mixed modeling [44]. Intracluster correlations were calculated at each time point to examine the potential effect of class clusters on the primary outcome, self-report weekly MVPA minutes. Given the small intracluster correlation values, the clustering (ie, classes) was not included in the linear mixed modeling.

To assess the impact of time, group, and group by time on the m-GLTEQ weekly MVPA minutes, linear mixed modeling was used via the *lme4* package in R (R Foundation for Statistical Computing) [45]. This approach was chosen because of its ability to handle unequal group sizes and retain participants with partial data (lost to follow-up). The models included fixed effects for group, time, group by time, and demographic variables with significant between-group differences at baseline that were not balanced via randomization. As random effects, random intercepts were included for participants to account for individual variation. The initial models included data from the baseline, 12-week, and 24-week time points. In line with the primary aim of the study, modeling was repeated using only the 12- and 24-week self-reported weekly MVPA data to further examine PA patterns during the PA maintenance period. Primary analyses followed intention-to-treat principles, with all the available data included in the models. To explore whether the extent of self-monitoring via Zamplo impacted intervention effectiveness (PA maintenance) relative to the waitlist control group, linear mixed modeling was repeated after splitting the intervention group using a tertile split according to participant app use in weeks (highest, middle, and lowest thirds). Sensitivity analyses were performed to determine the robustness of the modeling results to the impact of outliers and intervention noncompliance [46]. *P* values were obtained from the linear mixed models using Wald *F* tests with Satterthwaite approximation for denominator *df*. For the exploratory analyses of objective MVPA minutes and steps, the *P* values obtained from unpaired 2-tailed *t* tests were used to check for between-group differences at each week. Statistical significance was defined as *P*<.05 a priori.

## Results

### Recruitment and Study Completion

**Figure 1:**
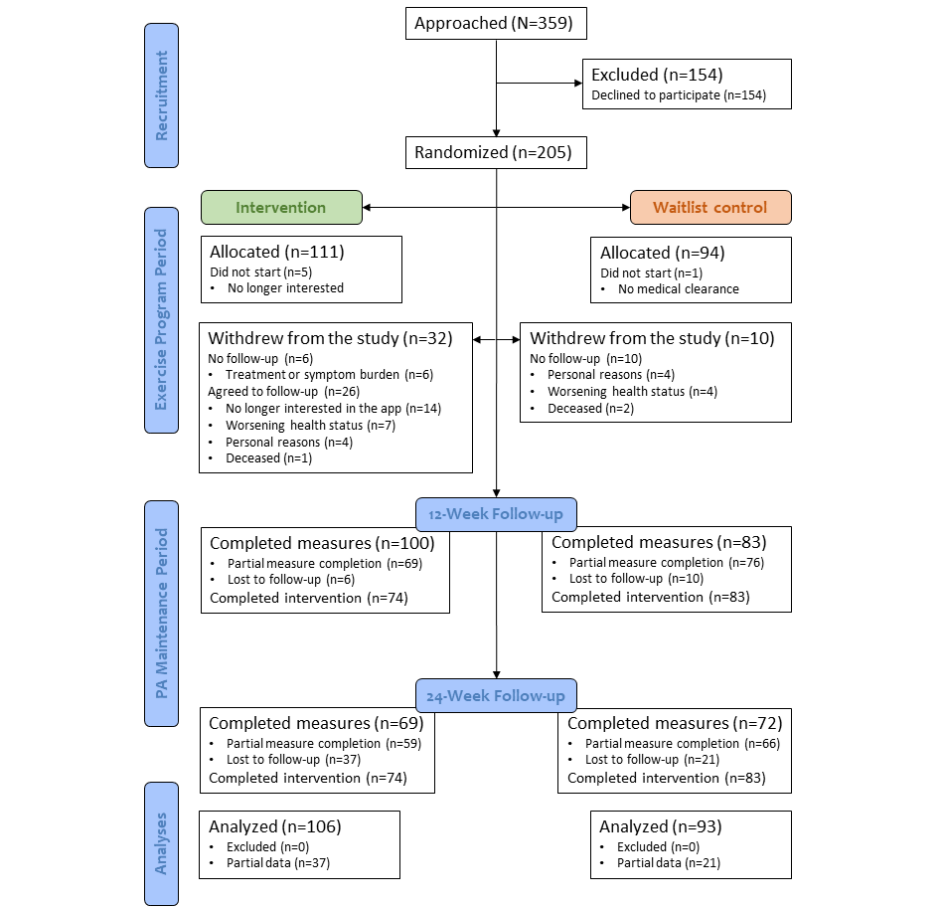
CONSORT (Consolidated Standards of Reporting Trials) diagram for the flow of participants through the study. PA: physical activity.

Details on participant flow throughout the study, including reasons for withdrawal, can be found in Figure 1. After 4 recruitment rounds over a 1-year period, 359 eligible EXCEL participants were approached, and 205 (57.1%) provided informed consent to participate in this study. Cluster randomization by EXCEL class resulted in 54.1% (111/205) of participants allocated to the intervention group and 45.9% (94/205) of participants allocated to the waitlist control group across 36 class clusters (intervention: n=18, 50%; waitlist control: n=18, 50%). A total of 6 participants (intervention: n=5, 83%; waitlist control: n=1, 17%) did not start this study, either owing to loss of interest or inability to obtain timely medical clearance. Over the first 12 weeks, 20.5% (42/205) of participants withdrew from the study. No withdrawals occurred between weeks 12 and 24. Of the 106 participants in the intervention group, 100 (94.3%) and 69 (65.1%) completed questionnaires at the 12- and 24-week time points, respectively. Of the 93 waitlist control participants, 83 (89%) and 72 (77%) completed follow-up questionnaires at the 12- and 24-week time points, respectively.

### Participants

Participant demographics are summarized in Table 2. The mean age of the study participants was 57.3 (SD 11.5) years (intervention: mean 56.7, SD 11.4 years; waitlist control: mean 58.0, SD 11.6 years; Table 2). All Canadian provinces and territories, except Nunavut, were represented by the participant population, with one-third (68/199, 34.2%) from Ontario. Most participants were female (174/199, 87.4%), White (163/199, 81.9%), and diagnosed with breast cancer (108/199, 54.3%). Other common cancer types included lung (24/199, 12.1%) and digestive (15/199, 7.5%) cancers. No significant between-group differences were found in age, sex, ethnicity, marital status, or employment status (all *P*>.05). However, the intervention group featured participants who were more educated (*P*=.01) and had higher incomes (*P*=.02) than those in the waitlist control group. Median m-GLTEQ weekly MVPA minutes at baseline were 60.0 (IQR 0-180) and 40.0 (IQR 0-135) for the intervention and waitlist control groups, respectively (*P*=.74).

Participants reported moderate QoL (FACT-G total score: mean 74.78, SD 15.61) and fatigue (FACIT-F: mean 35.15, SD11.03) at baseline, with no differences between groups (Table S1 in Multimedia Appendix 1). Both participant groups had similarly low eHealth literacy and high technology use (intervention: mean 7.1, SD 2.0 out of 10; waitlist control: mean 6.7, SD 2.7 out of 10), including smartphone use (intervention: 98/106, 92.5%; waitlist control: 82/93, 88%). Median EXCEL exercise class attendance was 83.3% (IQR 69.9%-95.7%) for the intervention group and 87.0% (IQR 71.3%-92.0%) for the waitlist control group (*P*=.80).

Compared with those who completed the study, participants who withdrew from the study were more likely to be male, widowed, or divorced; lack a university education; have a lower income; be off treatment; and report lower MVPA minutes at baseline (all *P*<.001).

**Table 2:** Participant baseline demographics.

						Total (n=199)			INT^a^ (n=106)			CTR^b^ (n=93)			*P* value^c^	
**Demographics**																
	Age (years), median (IQR)				59.0 (48.0-67.0)			58.0 (47.0-65.0)			61.0 (49.3-67.0)			.44		
	**Sex, n (%)**															.12
		Female		174 (87.4)			91 (85.8)			83 (89.2)						
		Male		25 (12.6)			15 (14.2)			10 (10.8)						
	**Ethnicity, n (%)**															.08
		East or Southeast Asian		8 (4)			6 (5.7)			2 (2.2)						
		Southern Asian		9 (4.5)			7 (6.6)			2 (2.2)						
		White		163 (81.9)			81 (76.4)			82 (88.2)						
		Other		24 (12.1)			17 (16)			7 (7.5)						
	**Location, n (%)**															N/A^d^
		Ontario		68 (34.2)			48 (45.3)			20 (21.5)						
		Saskatchewan		38 (19.1)			13 (12.3)			25 (26.9)						
		Nova Scotia		28 (14.1)			13 (12.3)			15 (16.1)						
		British Columbia		24 (12.1)			10 (9.4)			14 (15.1)						
		Alberta		16 (8)			7 (6.6)			9 (9.7)						
		New Brunswick		9 (4.5)			6 (5.7)			3 (3.2)						
		Manitoba		8 (4)			3 (2.8)			5 (5.4)						
		Other		8 (4)			6 (5.7)			2 (2.2)						
	**Income (CAD $^e^), n (%)**															.02
		<20,000		7 (3.5)			3 (2.8)			4 (4.3)						
		20,000-39,999		18 (9)			8 (7.5)			10 (10.8)						
		40,000-59,999		21 (10.6)			14 (13.2)			7 (7.5)						
		60,000-79,999		32 (16.1)			13 (12.3)			19 (20.4)						
		80,000-100,000		28 (14.1)			15 (14.2)			13 (14)						
		>100,000		72 (36.2)			44 (41.5)			28 (30.1)						
		Not disclosed		21 (10.6)			9 (8.5)			12 (12.9)						
	**Education, n (%)**															.01
		Some high school		0 (0)			0 (0)			0 (0)						
		Completed high school		13 (6.5)			5 (4.7)			8 (8.6)						
		Some university or college		37 (18.6)			15 (14.2)			22 (23.7)						
		Completed university or college		96 (48.2)			52 (49.1)			44 (47.3)						
		Some graduate school		3 (1.5)			2 (1.9)			1 (1.1)						
		Completed graduate school		50 (25.1)			32 (30.2)			18 (19.4)						
	**Marital status, n (%)**															.57
		Never married		11 (5.5)			6 (5.7)			5 (5.4)						
		Married		133 (66.8)			69 (65.1)			64 (68.8)						
		Common law		23 (11.6)			11 (10.4)			12 (12.9)						
		Separated		5 (2.5)			3 (2.8)			2 (2.2)						
		Widowed		11 (5.5)			7 (6.6)			4 (4.3)						
		Divorced		16 (8)			10 (9.4)			6 (6.5)						
	**Employment status, n (%)**															.06
		Disability leave		57 (28.6)			36 (34)			21 (22.6)						
		Retired		65 (32.7)			28 (26.4)			37 (39.8)						
		Part time		16 (8)			9 (8.5)			7 (7.5)						
		Homemaker		6 (3)			3 (2.8)			3 (3.2)						
		Full time		46 (23.1)			25 (23.6)			21 (22.6)						
		Temporarily unemployed		9 (4.5)			5 (4.7)			4 (4.3)						
	**Self-report weekly PA^f^ (m-GLTEQ^g^), median (IQR)**															
		MVPA^h^ minutes		45.0 (0-150.0)			60.0 (0-180.0)			40.0 (0-135.0)			.74			
		Resistance PA minutes		0 (0-21.25)			0 (0-15.0)			0 (0-30.0)			.21			
**Cancer characteristics**																
	**Cancer type, n (%)**															N/A
		Breast		108 (54.3)			54 (50.9)			54 (58.1)						
		Lung		24 (12.1)			12 (11.3)			12 (12.9)						
		Digestive		15 (7.5)			10 (9.4)			5 (5.4)						
		Gynecological		14 (7)			9 (8.5)			5 (5.4)						
		Genitourinary		12 (6)			8 (7.5)			4 (4.3)						
		Other		26 (13.1)			13 (12.3)			13 (14)						
	Advanced cancer, n (%)				47 (23.6)			25 (23.6)			22 (23.7)			N/A		
	**Current treatment, n (%)**															
		**Status**													N/A	
			On treatment	108 (54.3)			61 (57.5)			47 (50.5)						
			After treatment	91 (45.7)			45 (42.5)			46 (49.5)						
		**Type**													N/A	
			Surgery	38 (19.1)			21 (19.8)			17 (18.3)						
			Chemotherapy	12 (6)			8 (7.5)			4 (4.3)						
			Radiation	42 (21.1)			21 (19.8)			21 (22.6)						
			Hormone therapy	2 (1)			2 (1.9)			0 (0)						
			Biological therapy	34 (17.1)			24 (22.6)			10 (10.8)						
			Other	34 (17.1)			24 (22.6)			10 (10.8)						

^a^INT: intervention.

^b^CTR: waitlist control.

^c^*P* values were estimated using independent 2-tailed *t* tests for continuous variables and chi-square tests for categorical variables.

^d^N/A: not applicable.

^e^A currency exchange rate of CAD $1=US $0.74 is applicable.

^f^PA: physical activity.

^g^m-GLTEQ: modified Godin Leisure Time Exercise Questionnaire.

^h^MVPA: moderate-to-vigorous physical activity.

### Adherence to the Intervention Components: App Use and App Support Provided

App use information and a summary of the technical support provided for the app are presented in Table 3 and Figure S1 in Multimedia Appendix 1. Upon downloading the app in week 2, intervention group participants used the app for a median of 10.3 (IQR 1.3-22.9) weeks of a possible 23 weeks during the study period, with 52% (47/90) of participants using the app for at least 12 weeks. Approximately two-thirds (mean 66.3%, SD 37.2%) of app use was via a mobile device. Attendances at the first and second introductory workshops were 66% (70/106) and 50% (53/106), respectively. Participants reported 45 technical issues requiring 25.6 total hours of study team support to resolve.

**Table 3:** App use, workshop attendance, and app support provided.

		INT^a^ (n=106)
**App use, median (IQR)**		
	Weeks (out of 23)	10.3 (1.3-22.9)
	Entries per week	9.6 (4.4-17.8)
	Activities per week	9.5 (3.4-19.7)
	Symptoms tracked per week	20.4 (7.8-42.0)
**Introductory workshop attendance, n (%)**		
	Workshop 1	70 (66)
	Workshop 2	53 (50)
**Technical issues reported (n=45), n (%)**		
	Resolved via email	24 (53.3)
	Resolved via Zoom	21 (46.7)
**Time required to resolve**		
	Total hours	25.6
	Total minutes	1535
	Minutes per issue	34.1
	Minutes per user	14.5

^a^INT: intervention group.

### Primary Outcome: Self-Report MVPA Minutes

**Figure 2:**
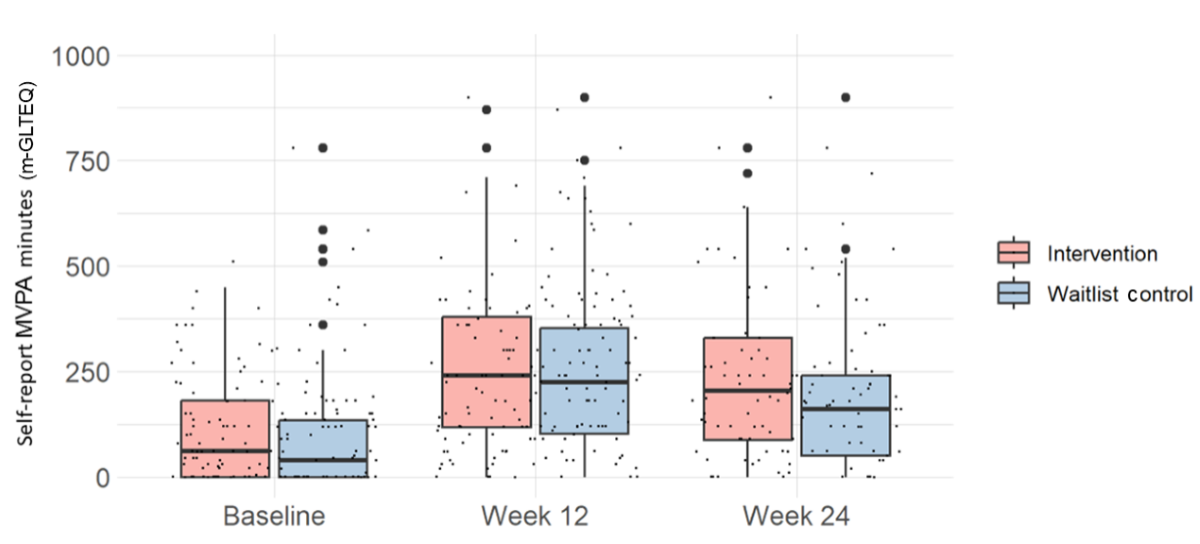
Boxplot of self-reported weekly moderate-to-vigorous physical activity (MVPA) minutes at baseline, week 12, and week 24. Black dots represent individual participants. m-GLTEQ: modified Godin Leisure Time Exercise Questionnaire.

Self-reported weekly MVPA minutes at baseline, week 12 (after EXCEL), and week 24 (after PA maintenance period) are shown in Figure 2 and Table 4. In the intervention group, median MVPA minutes per week were 60.0 (IQR 0.0-180.0) at baseline, 240.0 (IQR 117.5-378.75) at week 12, and 205.0 (87.5-330.0) at week 24. In the waitlist control group, median MVPA minutes per week were 40.0 (IQR 0.0-135.0) at baseline, 225.0 (IQR 102.5-352.5) at week 12, and 160 (IQR 55.0-180.0) at week 24. There were no between-group differences in weekly MVPA minutes at any time point (Table 4; *P*=.64-.90).

**Table 4:** Self-reported weekly moderate-to-vigorous physical activity minutes of the participants (n=199) at baseline, week 12, and week 24.

Time point	Intervention		Waitlist control		*P* value^a^
	Values, median (IQR)	Values, n (%)	Values, median (IQR)	Values, n (%)	
Baseline	60.0 (0.0-180.0)	105 (52.8)	40.0 (0.0-135.0)	91 (45.7)	.90
Week 12	240.0 (117.5-378.75)	96 (48.2)	225.0 (102.5-352.5)	82 (41.2)	.66
Week 24	205.0 (87.5-330.0)	68 (34.2)	160.0 (55.0-180.0)	71 (35.7)	.64

^a^*P* values for between-group differences at baseline, 12, and 24 weeks were calculated using unpaired 2-tailed *t* tests. *P* values <.05 were considered statistically significant as per the a priori cutoff.

Analyses via linear mixed modeling using data from all time points (Table 5) indicated a significant effect of time (week 12: mean 90.5%, SD 11.6% increase in self-report weekly MVPA minutes relative to baseline; week 24: 66.5%, SD 12.3% increase in self-report weekly MVPA minutes relative to baseline; *F*_2,289_=65.8; *P*<.001) but not group (*F*_1,163_=0.09; *P*=.76) or group by time (*F*_2,289_=0.14; *P*=.87). Education and income were included as fixed effects in the model to control for significant between-group baseline differences in these factors. No other demographic factors showed strong correlations with self-report weekly MVPA minutes to warrant inclusion in the model. A second linear mixed model focusing on the PA maintenance period between 12 and 24 weeks showed similar results, with a significant overall effect of time (week 24: mean −26.0%, SD 11.4% decrease in self-report MVPA minutes per week relative to week 12; *F*_1,140_=7.78; *P*=.006) but not group (*F*_1,147_=0.22; *P*=.64) or group by time (*F*_1,139_=0.26; *P*=.61; Table S2 in Multimedia Appendix 1). Exploratory analyses, with intervention group participants stratified by weeks of app use (highest, middle, and lowest thirds), indicated that intervention effectiveness did not differ between user subgroups (Table S2 in Multimedia Appendix 1). Sensitivity analyses confirmed that the mixed modeling results were robust to the presence of outliers and intervention noncompliance. The intracluster correlation of MVPA minutes with exercise class cluster was 0.008 at baseline, 0.025 at week 12, and 0.011 at week 24, indicating the limited effects of clustering (ie, classes) on the primary outcome.

**Table 5:** Linear mixed modeling results for weeks 0-24 (full intervention period)a.

	*F* test (*df*)	*P* value^b^
Group	0.09 (1,163)	.76
Time	65.8 (2,289)	<.001
Education	1.19 (4,162)	.32
Income	1.16 (5,164)	.33
Group × time	0.14 (2,289)	.87

^a^Logarithm (modified Godin Leisure Time Exercise Questionnaire moderate-to-vigorous physical activity minutes) ~ group × time + education + income + (1|participant).

^b^All *P* values were calculated via the linear mixed model using Wald *F* tests with Satterthwaite approximation for denominator df. *P* values <.05 were considered statistically significant as per the a priori cutoff.

### Secondary and Exploratory Outcomes

#### Mobile App Usability Ratings

**Figure 3:**
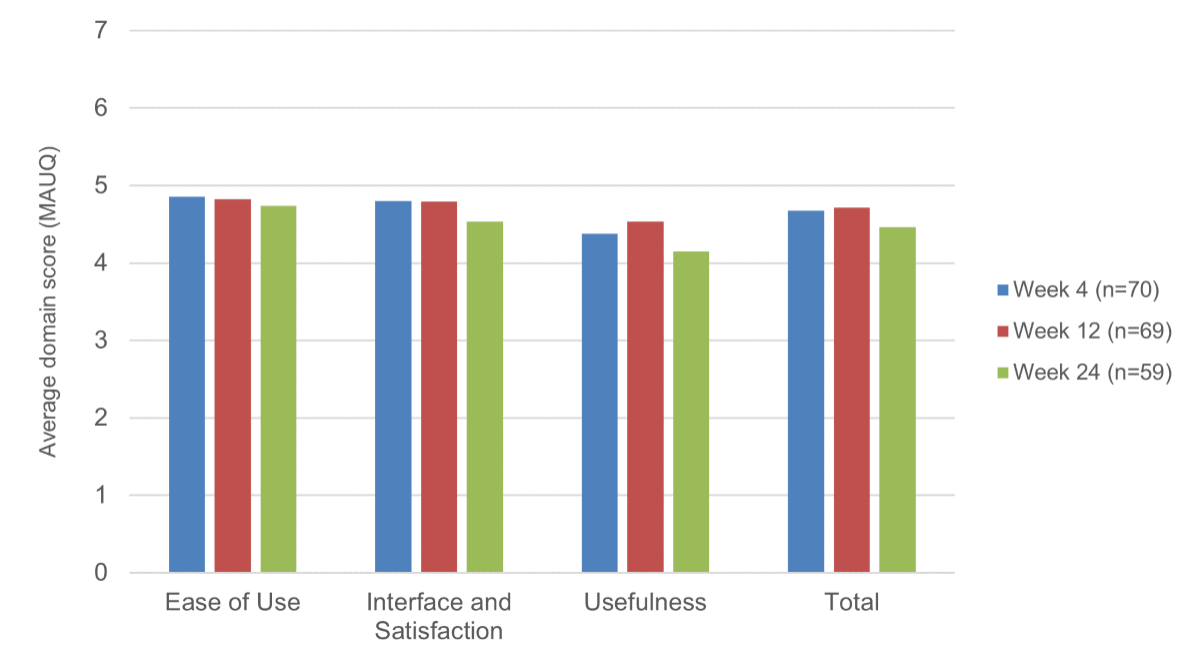
Participant-reported Mobile App Usability Questionnaire (MAUQ) scores over time.

Figure 3 summarizes the MAUQ ratings for the app at 3 time points: first impressions at week 4, after the EXCEL exercise program period at week 12, and after the PA maintenance period at week 24. On average, participants gave Zamplo moderate ease of use (4.7-4.9 out of 7), interface and satisfaction (4.5-4.8 out of 7), and usefulness (4.2-4.5 out of 7) scores.

#### Objective MVPA Minutes and Daily Steps

Owing to resource constraints, objective Garmin PA data were collected only for 55.3% (110/199) of participants, with 48.2% (96/199) recording valid data for an average of 14.7 (SD 9.1) weeks (intervention: average 16.8, SD7.9; waitlist control: average 13.1, SD 9.6). Of these, 46% (44/96) were in the intervention group, whereas the remaining 54% (52/96) were in the control group. Among this participant subset, the intervention and control groups did not differ with regard to demographics, except for higher income in the intervention group (*P*=.03); nor did they differ on baseline m-GLTEQ MVPA minutes (*P*=.80). An overview of the group averages for objective MVPA minutes and steps measured each week during the study period is provided in Figure S2 in Multimedia Appendix 1. Average daily steps during the 12-week exercise class period were 7345 (SD 2888) and 6219 (SD 2960) for the intervention and control groups, respectively. During the PA maintenance period (weeks 12-24), the average daily steps were 7995 (SD 2876) for the intervention group and 6159 (SD 2954) for the control group. There were no significant differences between groups for Garmin daily steps during the first 5 weeks. However, the intervention group had significantly higher daily steps than the waitlist control group during weeks 6 to 7, 9 to 12, 14 to 18, 20 to 21, and 23 (*P*=.048 to <.001; Figure S2 in Multimedia Appendix 1). During these weeks, the average daily steps in the intervention group were between 1200 and 3010 steps higher than those in the waitlist control group. There were no significant differences in Garmin MVPA minutes at any time point.

## Discussion

### Principal Findings

This study provides novel insights into the effectiveness of an app-based self-monitoring intervention in supporting PA maintenance after a 12-week exercise oncology program among rural and remote individuals living with and beyond cancer. Most previous eHealth exercise oncology interventions have recruited urban populations and did not examine postintervention PA maintenance [23]. Study participants in both the intervention and waitlist control groups increased their self-report weekly MVPA minutes directly after the 12-week exercise program and maintained significant increases at 24 weeks relative to baseline, indicating the positive impact of the EXCEL exercise oncology program. Additional support via the self-monitoring app did not improve self-report weekly MVPA during the PA maintenance period. Exploratory analyses indicated that app use may have contributed to significantly higher step counts during the later stages of the exercise class period and over half of the PA maintenance period.

Although the app included behavior change techniques (eg, self-monitoring and goal setting) linked to effective PA behavior change in oncology [8,10,47], its lack of additive impact on PA within this study may have been in part due to the effects of the EXCEL exercise program, which includes behavior change components that the app largely modeled. In addition, there was decreased app use, especially during the PA maintenance period. Specifically, the EXCEL exercise and educate program features behavior change techniques such as goal setting and barrier management, which were sufficient for supporting PA maintenance at 3 months after EXCEL [24]. Thus, there may have been limited potential for the self-monitoring app to further improve PA maintenance, especially within the first 12 weeks after EXCEL.

These results differ from some prior eHealth PA maintenance interventions for populations with cancer [48,49]. For example, an intervention containing telephone-based health coaching and tailored SMS text messages after an exercise oncology intervention was shown to improve PA maintenance [48]. However, the more intensive the health coaching and text messaging intervention was, the shorter the maintenance period was, and a lack of PA maintenance in the control group (highlighting potential differences in the effectiveness of the initial exercise programs provided by these studies to support PA maintenance) in the study by Gell et al [48] may contribute to the contrasting findings. However, other technology-based PA interventions in oncology also reported no significant intervention effects on PA maintenance, despite using a combination of technology and other supports (eg, phone counseling and printed materials) [50,51]. Research to date highlights that more resource-intensive interventions are not always better, with varied individual needs and preferences for eHealth PA maintenance support.

Notably, nearly 50% of the intervention group participants in this study stopped using the app before the PA maintenance period, indicating significant ease of use challenges, a lack of perceived value, or both [52]. For individuals with prior PA experience, as was the case for many participants in the present sample, and those who are already receiving behavior change support within EXCEL, the use of a self-monitoring app such as Zamplo may have limited utility. Research shows that the continued use of eHealth in behavior change interventions is driven by participants’ perceived value of the intervention [53]. In addition, low eHealth literacy scores among participants may have led to greater challenges with using the app. App improvements that are tailored to meet user needs and integrate evidence-based PA maintenance behavior change techniques (eg, graded tasks and action planning) may enhance intervention engagement and potentiate the intervention’s effectiveness in supporting PA maintenance [10]. For example, although the app included the ability to chat one to one with other participants, further social functionality (eg, team PA challenges and group messages) may improve app engagement and support behavior change [54,55]. Tailoring and optimizing eHealth components will be especially important for interventions targeting rural and underserved populations, who often face greater PA barriers, including less social support [56].

Whereas no intervention effects on MVPA maintenance were observed, daily step counts collected via Garmin devices were significantly higher in the intervention group for extended periods, including more than half of the PA maintenance period. This points to a potential positive impact of app-based self-monitoring on daily steps. These effects may be clinically relevant, exceeding the minimal important difference of approximately 1000 steps noted in prior chronic disease research [57,58]. Although MVPA is typically associated with greater health benefits, increased step counts may also contribute to improvements in physical and psychosocial well-being in oncology [1,59,60]. These findings speak to the value of measuring PA across varying intensities and using both objective and subjective PA measures to comprehensively examine the potential effects of technology-supported exercise oncology interventions. However, positive intervention effects on daily steps can only be seen as preliminary, given that objective PA data were not captured for all participants and that data availability was greatly reduced during the final weeks of the PA maintenance period.

### Strengths and Limitations

This study has several strengths, including a large sample size coupled with a linear mixed modeling approach, leading to robust analyses of intervention effectiveness for supporting PA maintenance based on all the available data. Given the smaller sample sizes, single-arm designs, and limited measurement of PA maintenance in many previous eHealth exercise oncology intervention studies, this study adds significantly to the existing literature [23]. However, the participant sample was biased toward a subset of the population with cancer with above-average baseline PA, well-being, and socioeconomic status, with an overrepresentation of White female patients with a breast cancer diagnosis. Therefore, the results of this study may not be generalizable to other populations with cancer. Although the measurement of subjective and objective PAs painted a more comprehensive picture of PA behaviors herein, PA self-reporting is prone to recall and social desirability biases, and only a subset of participants received trackers owing to financial and logistical constraints. Furthermore, despite the intracluster correlations indicating no significant effects of class clusters on the primary outcome, future work may consider randomly assigning participants to class sites to reduce potential selection bias. In this EXCEL effectiveness-implementation trial, this level of randomization was not possible, as participants joined web-based class sites based on geographic location and class timing preferences. These are important considerations for interpreting the PA outcomes of this study. Finally, the selected app was designed for populations with cancer and tailored to participant needs via user-centered codevelopment [28]. The codevelopment process also prompted the integration of behavior change techniques that are linked to effective PA behavior change (eg, prompts or cues and feedback) into the app [28,29,47,61]. Tailoring technology to participant needs and integrating evidence-based behavior change techniques are valuable steps to enhance engagement and potential effectiveness in eHealth interventions [61-63]. Despite this theory-informed co-design process, using an existing app limited the customizability of the tracking experience specific to PA. As highlighted by the technical issues, decreased use over time, and lack of effects on MVPA maintenance, further improvements to the existing app or the development of newer, more effective mobile apps or other eHealth tools may be required to better support PA maintenance in this participant population.

### Future Work

Qualitative data from participant interviews conducted at 24 weeks were used to better understand participants’ perspectives on the ease of use and potential value of the current self-monitoring app to support PA maintenance. These results will provide further insights into the potential impact of app-based self-monitoring in exercise oncology, address important research gaps (ie, what works, for whom, and why), and help inform future eHealth exercise oncology interventions.

Beyond this study, additional work is needed to examine the potential of eHealth for exercise maintenance. Given the limited impact of the present app, future eHealth exercise oncology studies may want to use other PA-specific apps such as WalkOn, which has been shown to increase weekly steps among individuals with breast cancer, or Heal-Me, an app designed to support health behavior change among older populations living with chronic diseases [64,65]. Future research can leverage alternative trial designs (eg, sequential multiple assignment randomized trials and preference-based trials) to build knowledge on what eHealth interventions best support PA maintenance, as well as the *how* and *why* of their effectiveness [7,66,67]. The results of such studies can inform the development of tailored eHealth interventions for supporting PA maintenance across various populations with cancer. Importantly, codevelopment with potential users, to maximize the ease of use and personal relevance, and researchers, to ensure that the technology draws upon the best evidence in PA maintenance, is recommended to develop highly effective eHealth tools [68-71].

### Conclusions

In this study, a self-monitoring app-based intervention did not improve MVPA maintenance among remote and rural populations with cancer after they completed a supervised web-based exercise oncology program. Participants in both the intervention and waitlist control groups maintained significant increases in MVPA at 24 weeks, indicating that the 12-week EXCEL exercise program alone supported MVPA maintenance in the present sample. Objective PA data from a subset of participants highlighted the potential positive effects of the app on daily steps during the PA maintenance period. Future work should examine the impact of eHealth on PA maintenance in those who may require more PA behavior change support. In addition, research is warranted on the long-term PA maintenance effectiveness (ie, beyond 6 months) and optimal components for eHealth exercise oncology interventions.

## Appendix

Multimedia Appendix 1 Additional baseline and outcome data.

Multimedia Appendix 2 CONSORT-eHEALTH checklist V 1.6.1.

## Abbreviations

eHLQ: eHealth Literacy Questionnaire
ESAS: Edmonton Symptom Assessment System
EXCEL: Exercise for Cancer to Enhance Living Well
FACIT-F: Functional Assessment of Chronic Illness Therapy-Fatigue
FACT-Cog: Functional Assessment of Cancer Therapy-Cognitive Function
FACT-G: Functional Assessment of Cancer Therapy-General
MAUQ: Mobile App Usability Questionnaire
m-GLTEQ: modified Godin Leisure Time Exercise Questionnaire
MVPA: moderate-to-vigorous physical activity
PA: physical activity
QoL: quality of life
RCT: randomized controlled trial
REDCap: Research Electronic Data Capture

## Data Availability

The data sets generated or analyzed during this study are available from the corresponding author upon reasonable request.
