# Supplementary material for: Effectiveness of a Self-Monitoring App in Supporting Physical Activity Maintenance Among Rural Canadians With Cancer After an Exercise Oncology Program: Cluster Randomized Controlled Trial
Source: JMIR Cancer. 2023 Sep 7;9:e47187. doi: 10.2196/47187 (PMC10514772; doi:10.2196/47187)

## Multimedia Appendix 1

Table S1: Baseline psychosocial characteristics, electronic health literacy, and technology use.^a^

|  |  | **Total (n=199)** | **INT (n=106)** | **CTR (n=93)** | ***P*-value** |
| --- | --- | --- | --- | --- | --- |
| **Psychosocial Characteristics** | |  |  |  |  |
| FACT-G, Mean (SD) | |  |  |  |  |
|  | Physical Well-being | 21.3 (4.8) | 21.2 (4.7) | 21.7 (5.0) | .22 |
|  | Social/Family Well-being | 18.7 (4.5) | 18.7 (4.6) | 19.1 (4.3) | .24 |
|  | Emotional Well-being | 17.4 (4.7) | 17.3 (5.0) | 18.0 (4.2) | .06 |
|  | Functional Well-being | 17.4 (5.7) | 17.4 (5.6) | 18.3 (5.7) | .05 |
|  | Total Well-being | 74.8 (15.6) | 74.6 (15.2) | 77.2 (15.9) | .05 |
| FACT-Cog, Mean (SD) | |  |  |  |  |
|  | Perceived cognitive impairments | 56.1 (18.8) | 56.2 (17.8) | 57.3 (20.0) | .42 |
|  | Comments from others | 14.5 (2.5) | 14.5 (2.2) | 14.5 (2.8) | .81 |
|  | Perceived cognitive abilities | 22.9 (8.1) | 22.9 (7.8) | 23.5 (8.4) | .31 |
|  | Impact on quality of life | 10.4 (4.7) | 10.4 (4.7) | 10.9 (4.6) | .16 |
| FACIT-Fatigue, Mean (SD) | |  |  |  |  |
|  | Total | 35.2 (11.0) | 35.0 (10.5) | 36.6 (11.5) | .10 |
| ESAS, Mean (SD) | |  |  |  |  |
|  | Pain | 2.2 (2.2) | 2.2 (2.3) | 2.0 (2.2) | .18 |
|  | Tiredness | 3.8 (2.5) | 3.8 (2.5) | 3.6 (2.5) | .35 |
|  | Drowsiness | 2.7 (2.7) | 2.8 (2.6) | 2.7 (2.7) | .84 |
|  | Nausea | 0.6 (1.5) | 0.6 (1.2) | 0.7 (1.8) | .22 |
|  | Lack of Appetite | 1.4 (2.4) | 1.3 (2.5) | 1.3 (2.2) | .57 |
|  | Shortness of Breath | 1.1 (2.1) | 1.1 (1.9) | 1.2 (2.2) | .31 |
|  | Depression | 1.8 (2.3) | 1.8 (2.4) | 1.6 (2.2) | .23 |
|  | Anxiety | 2.1 (2.4) | 2.1 (2.3) | 1.9 (2.2) | .34 |
|  | Wellbeing | 3.4 (2.4) | 3.4 (2.4) | 3.3 (2.4) | .63 |
|  | Total | 19.1 (13.7) | 19.1 (13.5) | 18.3 (14.0) | .49 |
| **Electronic Health Literacy (eHLQ), Mean (SD)** | | |  |  |  |
| Using technology to process health information | | 1.9 (0.5) | 1.9 (0.5) | 1.8 (0.6) |  |
| Understanding of health concepts and language | | 2.1 (0.4) | 2.1 (0.4) | 2.0 (0.5) |  |
| Ability to actively engage with digital services | | 2.0 (0.5) | 2.1 (0.4) | 1.9 (0.6) |  |
| Feel safe and in control | | 2.1 (0.5) | 2.1 (0.5) | 2.1 (0.5) |  |
| Motivated to engage with digital services | | 1.9 (0.5) | 2.0 (0.4) | 1.9 (0.6) |  |
| Access to digital services that work | | 1.7 (0.5) | 1.7 (0.5) | 1.7 (0.6) |  |
| Digital services that suit individual needs | | 1.6 (0.6) | 1.6 (0.5) | 1.6 (0.6) |  |
| **Technology Use** | |  |  |  |  |
| Total tech use /10, Mean (SD) | | 6.9 (2.4) | 7.1 (2.0) | 6.7 (2.7) |  |
| Computer/Laptop, No. (%) | | 169 (84.9) | 95 (89.6) | 74 (79.6) |  |
| Smartphone, No. (%) | | 180 (90.5) | 98 (92.5) | 82 (88.2) |  |
| Internet via Computer, No. (%) | | 165 (82.9) | 94 (88.7) | 71 (76.3) |  |
| Internet via Smartphone, No. (%) | | 164 (82.4) | 90 (84.9) | 74 (79.6) |  |
| PA/Health App, No. (%) | | 85 (42.7) | 46 (43.4) | 39 (41.9) |  |
| Wearable Activity Tracker, No. (%) | | 118 (59.3) | 59 (55.7) | 59 (63.4) |  |
| Tablet, No. (%) | | 127 (63.8) | 69 (65.1) | 58 (62.4) |  |
| Social Media, No. (%) | | 148 (74.4) | 82 (77.4) | 66 (71.0) |  |
| Email, No. (%) | | 183 (92.0) | 100 (94.3) | 83 (89.2) |  |
| Other, No. (%) | | 39 (19.6) | 24 (22.6) | 15 (16.1) |  |

^a^P-values were calculated using independent t-tests for continuous variables and chi-square tests for categorical variables.

Figure S1: Percentage of participants using Zamplo during the study period.^a^

^a^ The Zamplo platform was introduced at week 2 of the EXCEL exercise program.

Table S2: Results from the linear mixed models for the PA maintenance period between 12 and 24 weeks and the exploratory user sub-group analyses.

| **Linear mixed modeling results, Week 12-24 (maintenance period only)** | | | | |  |
| --- | --- | --- | --- | --- | --- |
| LOG(GLTEQ MVPA minutes) ~ Group*Time + Education + Income + (1\|Participant) | | | | | |
|  | **F-value** | **P-value** |  |  |  |
| Group | 0.22 | 0.64 |  |  |  |
| Time | 7.78 | 0.006 |  |  |  |
| Education | 0.94 | 0.44 |  |  |  |
| Income | 2.11 | 0.07 |  |  |  |
| Group*Time | 0.26 | 0.61 |  |  |  |
| **Linear mixed modeling results, sub-grouping stratified by actual Zamplo usage** | | | | |  |
| LOG(GLTEQ MVPA minutes) ~ Group*Time + Education + Income + (1\|Participant) | | | | | |
|  | **F-value** | **P-value** |  |  |  |
| Group | 0.055 | 0.95 |  |  |  |
| Time | 56.5 | <0.0001 |  |  |  |
| Education | 1.18 | 0.32 |  |  |  |
| Income | 1.13 | 0.34 |  |  |  |
| Group*Time | 0.69 | 0.6 |  |  |  |

Figure S2: Overview of objective PA data as measured via the Garmin Vivosmart 4.


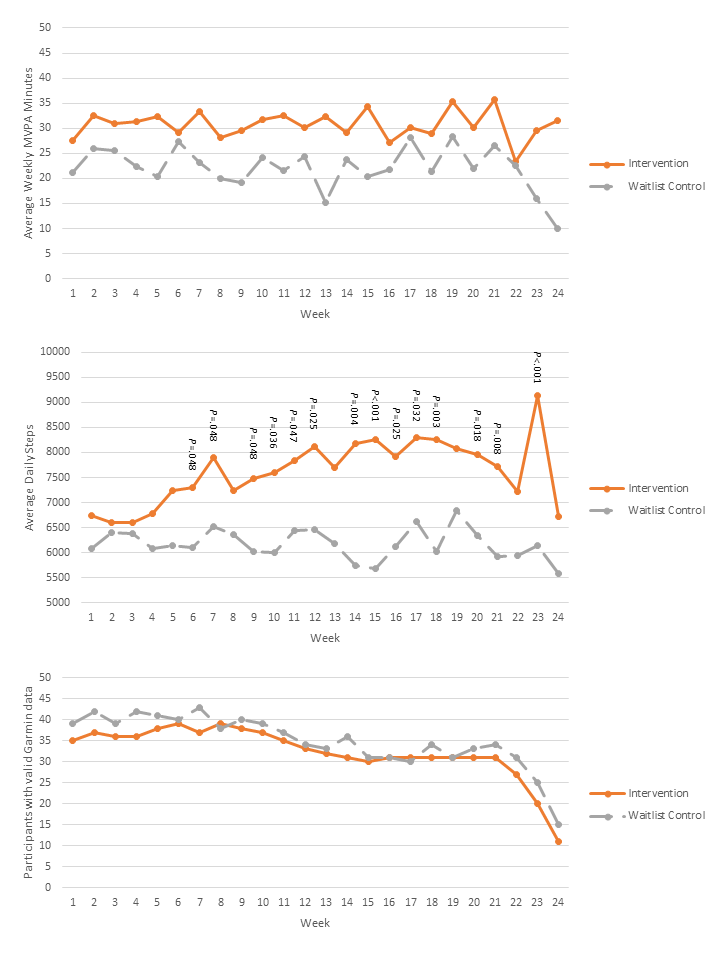

Supplement: Multimedia Appendix 1 [file cancer_v9i1e47187_app1.docx]
